# Piezoelectric Polymer and Paper Substrates: A Review

**DOI:** 10.3390/s18113605

**Published:** 2018-10-24

**Authors:** Kiran Kumar Sappati, Sharmistha Bhadra

**Affiliations:** Department of Electrical and Computer Engineering, McGill University, Montreal, QC H3A 0E9, Canada; sharmistha.bhadra@mcgill.ca

**Keywords:** piezoelectric, polymer, paper, sensors, substrates

## Abstract

Polymers and papers, which exhibit piezoelectricity, find a wide range of applications in the industry. Ever since the discovery of PVDF, piezo polymers and papers have been widely used for sensor and actuator design. The direct piezoelectric effect has been used for sensor design, whereas the inverse piezoelectric effect has been applied for actuator design. Piezo polymers and papers have the advantages of mechanical flexibility, lower fabrication cost and faster processing over commonly used piezoelectric materials, such as PZT, BaTiO_3_. In addition, many polymer and paper materials are considered biocompatible and can be used in bio applications. In the last 20 years, heterostructural materials, such as polymer composites and hybrid paper, have received a lot of attention since they combine the flexibility of polymer or paper, and excellent pyroelectric and piezoelectric properties of ceramics. This paper gives an overview of piezoelectric polymers and papers based on their operating principle. Main categories of piezoelectric polymers and papers are discussed with a focus on their materials and fabrication techniques. Applications of piezoelectric polymers and papers in different areas are also presented.

## 1. Introduction

Electromechanical transduction is the main principle of piezoelectricity. This feature is largely used to generate electrical signal in response to applied mechanical stress, with applications ranging from the flashing lights in children’s shoes to motion- or vibration-powered wireless sensors. A converse piezoelectric effect is also important, which gives mechanical output in response to applied electrical field by changing shape [1]. Furthermore, piezoelectric effect is the basis of several scientific instrumentation techniques with atomic resolution, such as scanning tunneling microscope (STM), Atomic force microscope (AFM), as well as everyday use devices, such as cigarette lighters, push–start propane barbecues, and quartz watches. The piezoelectric sensor basically converts changes in measurand to an electrical signal which is forwarded to an electronic circuit for further analysis. Piezoelectricity is realized in a wide variety of materials but only a few of have been used for sensors. Pressure sensors, flow sensors, vibration sensors, bio implantable devices, ultrasound devices, and gas sensors are some examples. Key properties of piezoelectric ceramics have made it possible to realize high-frequency resonant structures, low-power sensors with broad dynamic ranges, and large-amplitude actuators with lower driving voltage, hysteresis, and CMOS compatibility [2]. We will review important features of piezoelectric polymer and paper that made them prosperous in finding interesting applications, specifically for sensing.

A wide variety of piezoelectric materials are available to researchers and engineers. However, the search for improvement is never ending. Piezoelectric materials can be broadly classified as naturally occurring and synthetic. Quartz, Rochelle salt, Topaz, Tourmaline-group and few organic substances fall under the natural category and materials such as Barium titanate (BaTiO${}_{3}$), Lead titanate (PbTiO${}_{3}$), Lithium niobate (LiNbO${}_{3}$), and Lead zirconate titanate (PbZr${}_{x}$ Ti${}_{1-x}$O${}_{3}$, 0 < x < 1) more commonly known as PZT, and Zinc oxide (ZnO) are synthetic materials. Synthetic materials are also called piezoelectric ceramics. After the World War II, piezoelectric materials started gaining popularity due to the discovery of ceramics, mainly because of their ability to fine-tune the physical parameters such as piezoelectric constant, dielectric permitivity and stiffness according to the need [3]. This led to the huge success of piezoelectric ceramics in many applications. Extensive focus has been given in the last two decades to develop new piezoelectric compounds such as piezopolymers, piezo papers and lead free piezoelectric materials, resulting in major improvements in electromechanical coefficients, material properties, and application areas. The cleaner and greener drive for environment has made the researchers look for non-toxic materials e.g., without lead (Pb). For non-toxic piezo materials, many new materials using cellulose paper, Polyvinylidene diflouride (PVDF), polymer composites, Aluminum Nitride (AlN), etc., have emerged in the last few years [2]. Even though polymer and paper do not match the piezoelectric properties of ceramics, they have made their way into numerous applications, owing to their biocompatibility, biodegradability, flexibility, low cost, low power consumption and easy deployability.

Piezoelectric polymers and papers are crucial for delivering highly smart and integrated piezoelectric devices. Research for the compatible and readily adoptable materials for biomedical sensing applications is one of the ever-demanding areas for piezoelectric materials. Piezoelectric materials with high elastic modulus such as quartz have well established lithographic patterning, growth and deposition processes. However, these materials are hard and brittle, and may not be compatible with biomedical applications, despite their high-performance capabilities. Polymers and papers can fill this gap efficiently with the advancements in fabrication techniques such as laminate, dice and cut method and functionalizing paper with piezoelectric ceramic nano particles. It is also important to mention that synthesis of various nano structures and modalities of piezoelectric ceramics, such as ZnO, BaTiO${}_{3}$ and PZT, enabled conceiving attractive custom-made applications, such as nanogenerators and self powered sensors. Piezoelectric polymers and papers with multi layered heterostructures are manufactured using the standard micromachine process tools and can be readily deployable anywhere. In addition, some lead free, highly efficient composites are made with polymers and paper mixed with piezoelectric ceramics such as KNbO${}_{3}$, BaTiO${}_{3}$, and BaZrTiO${}_{3}$. These composites are suitable to bio medical applications. Unlike piezo ceramics and crystals, piezoelectric polymers have low acoustic impedance which makes them favorable for sensing in the environments like water, human tissue and other organic materials [4]. Paper has also been a unique choice in realizing some appealing applications as it is best suited for environmentally friendly and biocompatible products. Cellulose crystallites in the paper are primarily responsible for the shear piezoelectric constants which are comparable to quartz [5]. Polymers and papers are the best choices in making the piezoelectric films/substrates, as the processes of manufacturing are simple, low cost and uses low temperature and existing micro fabrication techniques. In this article, we have reviewed the materials, various fabrication techniques and applications of piezoelectric polymers and papers.

## 2. Piezoelectricity

Since the first reporting of direct piezoelectric effect by Piere and Jacque curie in 1880 and converse piezoelectric effect by Lippmann in 1881, no significant developments were made until the discovery of PZT and BaTiO${}_{3}$ in the 1950s. In 1971, Jaffe et al. discovered piezoelectric ceramics. Then, the use of piezoelectric materials in various applications picked up. In this section, we will discuss about the theory behind the phenomena of piezoelectricity and key performance characteristics related to these materials.

### 2.1. The Materials

About 30% of the material available in the world can exhibit piezoelectricity [6]. Even though a wide variety of materials show this property, only a few of them have found useful applications in science. Non-centro symmetry in materials is crucial in understanding this effect; at the same time, it is just a necessary but not sufficient condition. Except for cubic classes 432, all other non-centro symmetric crystallographic classes can display piezoelectricity. The piezoelectric materials can be broadly classified into [1]:

1. Single crystals: Quartz, LiNbO${}_{3}$, Lithium Tantalate (LiTaO${}_{3}$),
2. Poly crystalline materials: BaTiO${}_{3}$, PbTiO${}_{3}$, Lead Zirconate(PbZrO${}_{3}$),
3. Relaxator Ferro electrics: Lead Magnesium Niobate-Lead Titanate (PMN-PT), Lead Zirconium Niobate-Lead Titanate (PZN-PT),
4. Polymers: PVDF, Poly (vinylidene diflouride- trifluoro ethylene) P(VDF-TrFE), Polymer-Ceramic composites.
5. Piezoelectric Paper

It is important to note that man-made or synthetic piezoelectric ceramics, such as BaTiO${}_{3}$, PZT can perform far better than the natural ones, such as Quartz, Rochelle salt and Topaz, in terms of their desired electrical and mechanical properties [7]. For instance, Pb(Mg0.33 Nb0.67)O${}_{3}$-PbTiO${}_{3}$ (PMN-PT) has exceptional piezoelectric charge constant, $d_{33}$ which is in the order of 2500 pC/N, almost 1000 times higher than that of Quartz [8].

### 2.2. The Constitutive Equations

Equations (1) and (2) are used to describe the relation between electromechanical properties of the piezoelectric material. It is important to note that, at low electrical stress, the produced mechanical stress is linear, but, as the electrical stress increases, this effect tends to be nonlinear. However, most of the piezoelectric materials in sensing applications operate in the linear zone [9]:

(1) $$x_{i}=S_{ij}^{D}\sigma_{j}+d_{mj}E_{m},$$

(2) $$D_{m}=d_{mj}^{D}\sigma_{i}+\epsilon_{ik}^{\sigma }E_{k},$$

where *i*, *j* = 1, 2,…, 6 and *m*, *k* = 1, 2, 3 refer to different directions within the material coordinate system.

The most useful modification of Constitutive equations for sensors can be expressed as

(3) $$x_{i}=S_{ij}^{D}\sigma_{j}+g_{mi}D_{m},$$

(4) $$E_{i}=g_{mi}\sigma_{i}+\beta_{ik}^{\sigma }D_{k},$$

where 


σ
is Stress Vector (N/m${}^{2}$),
*x*
is Strain Vector (m/m),
*E*
is Applied Electrical field in (V/m),
ϵ
is permittivity in (F/m),
*d*
is piezoelectric constant (m/V),
*S*
is Matrix of compliance coefficients (m${}^{2}$/N),
*D*
is Electrical displacement (C/m${}^{2}$),
*g*
is piezoelectric constant (m${}^{2}$/C),
β
is impermittivity component (m/F).

**Figure 1:**
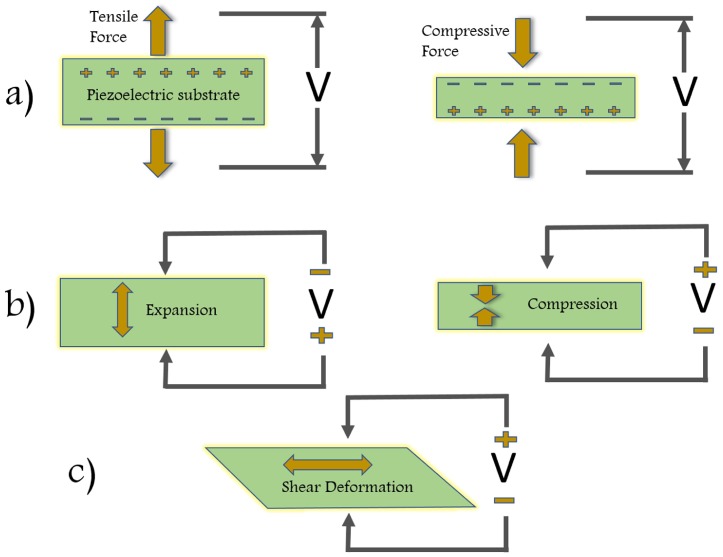
Schematic representation of the longitudinal direct (**a**); converse (**b**); and shear (**c**) piezoelectric effects.

Figure 1 explains all possible variations in electrical polarization, P with the changes in direction of applied mechanical stress. The ability of materials to develop electric displacement, D that is directly proportional to an applied mechanical stress is defined as piezoelectricity. There will be an opposite electric charge (reverses direction) if the stress is changed from tensile to compressive (Figure 1a). The converse effect of piezoelectricity is explained by Figure 1b, i.e., they deform under applied electric field and the direction of the deformation changes with the applied electric field direction. The effect of Shear piezoelectricity (Figure 1c) linearly couples shear mechanical stress or strain with the electric charge [7].

## 3. Polymer Based Substrates

### 3.1. Introduction

The practical limitation of piezoelectric ceramics is brittleness which can lead to accidental breakages. This made polymers a better choice in many applications. In the last two decades, piezoelectric polymers like PVDF, P (VDF-TrFE), poly propylene (PP) have been extensively explored for many applications, such as energy harvesting, sensing, actuation and bio medical fields. Details of piezoelectric polymer applications will be reviewed in the Applications section later. In this section, we will focus on some key factors of piezoelectric polymers, such as classification, materials and fabrication methods. Table 1 and Table 2 illustrate the comparison of performance factors of different piezoelectric materials including piezoelectric ceramics, polymers (bulk) and polymer composites.

### 3.2. Classification

Classification of piezoelectric polymers is based on the topology and dipole moment. They are divided into bulk polymers, polymer composites and voided charged polymers. Bulk polymers exhibit a piezoelectric effect depending on their molecular structure. Polymer composites are made of piezoelectric ceramic nano particles combined with polymers like PVDF, PDMS (poly dimethyl siloxane), and PP. The piezoelectric effect in these materials is basically imparted by the piezoelectric ceramic particles and flexibility to the composite is introduced by polymers. Voided charged polymers (VCP) are a different type of thin polymer films with gas voids made in it. These gas voids are charged by an application of an electrical field to form internal dipoles which are responsible for a piezoelectric effect.

**Figure 2:**
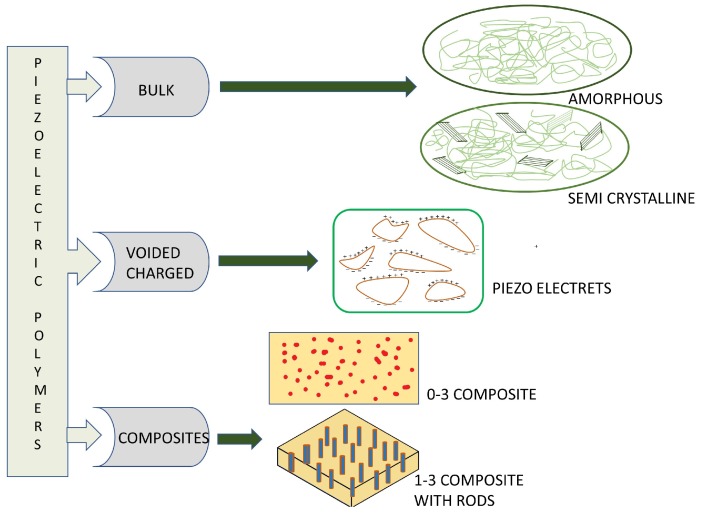
Classification of piezoelectric polymers: bulk piezo polymers, voided charged polymers or piezoelectrets and piezoelectric polymer composites.

Figure 2 depicts the structural details of various piezoelectric polymers explained above. The morphology in the amorphous regions decides the thermal operation of the polymer and the polar crystalline phases dispersion are responsible for the piezoelectric effect. The characterization of piezoelectric polymers involves quasistatic or direct techniques. Amorphous polymers use direct techniques and semicrystalline polymers employ thermally simulated current (TSC) measurements for the same parameters, as current and remnant polarization are functions of temperatures.

#### 3.2.1. Bulk Polymers

In theory, ferroelectricity is largely dependent on the crystallinity of the material meaning only crystalline or semicrystalline materials can be possible ferroelectrics materials. Therefore, it is important to understand the molecular structures, also called crystallanity of the polymer, which exhibit piezoelectricity. Degree of crystallinity is decided by semicrystalline and amorphous regions. However, ferroelectricity in amorphous polymers lacking crystalline structure is also proven by researchers. Molecular dipoles in the amorphous regions largely contribute to the piezoelectric effect. Crucial thermal properties such as glass transition temperature, $T_{g}$ is defined by the amorphous regions and the melting point, and $T_{m}$ of the polymer is decided by the crystalline regions. The degree of crystallinity in these materials is dependent on the method of preparation and thermal history. Based on the molecular structure, these polymers can be divided into two groups: semi crystalline and amorphous.

Semicrystalline structures are not of a single crystal structure; rather, they are formed by randomly oriented microscopic crystals distributed within an amorphous bulk. Mechanical orientation, thermal annealing and high voltage treatment can be used to induce crystalline phase transformations. Stretching the polymer hugely helps in aligning the amorphous strands in the film plane and facilitates for the uniform rotation of the crystallites by an electric field [12]. PVDF and its copolymers, Polyamides, and Paralyne-C come under this category. PVDF is a special polymer which has many crystalline forms in which β-phase creates a high piezoelectric effect. Special efforts are made to improve the β-phase content in PVDF. Soin et al. tried to improve β-phase content in PVDF by a phase inversion technique and controlling the quenching temperature [13]. Poling is needed for semicrystalline materials to re-orient the crystallites for enhancing the piezoelectric effect and aligning the molecular dipoles. As in the piezoelectric inorganic materials, the equivalent centre of charge displacement causes a change in polarization by the presence of an applied electrical field in polymers.

Another interesting classification of bulk polymers is amorphous, which does not contain a long range order to support the piezoelectric effect. Non-crystalline materials such as Polyimide and PVDC come under this category. To get the alignment of the macroscopic crystals in these polymers, poling is needed near the $T_{g}$, which is less compared to semicrystalline polymers. Piezoelectricity in amorphous polymers is largely dependent on the orientation of molecular dipoles. Unlike semicrystalline polymers, the polarization is quasi stable due to freezing of molecular dipoles. Hence, the poling factor is important in net piezoelectricity. Poling is done at slightly above $T_{g}$, which ensures the effective orientation and locking of the molecular dipoles. In addition, poling affects the piezoelectric coefficient of amorphous polymers hugely. For example, piezoelectric coefficient of PVC improves by 4–5 times after poling. An important thing to note is, after cooling, these dipoles are not in thermal equilibrium in contrary to semicrystalline polymers. The resultant piezoelectric constant, $d_{31}$ can be expressed using remnant polarization, $P_{r}$ and poling electrical field $E_{p}$:

(5) $$d_{31}=P_{r}\left(1-\gamma \right)S_{11}+\frac{P_{r}\left(1-\gamma \right)}{3}\left(\epsilon_{\infty }\right)-1)S_{11}.$$

here, 


γ
is the Poisson ratio,
$S_{11}$
is the compliance,
$\epsilon_{\infty }$
is the permittivity at higher frequencies.

#### 3.2.2. Piezoelectric Composites

Ceramic/polymer composite can be defined as a material in which the ceramic phase is dispersed in a polymer matrix [14]. Mechanical properties of polymers make them reaching the application areas where traditional piezoelectric ceramic crystals are not effective such as flexible and wearable electronics. They have much higher piezoelectric stress constant, *g* leading to better sensors than ceramics [12]. Typical ceramic material is made of a metal, non-metal, or a metalloid bonded by means of ionic or covalent interactions, popular due to their high mechanical strength, thermal and chemical stability, elastic modulus, and wear resistance. Piezoelectric ceramic particles such as BaTiO${}_{3}$, PZT, PMN-PT, ZnO which are of micro and nano dimensions are employed in polymer nanocomposites (nPC). Piezocomposites can be made by simple blending of piezoelectric ceramics and polymers, but the connectivity patterns of these constituents control the electric flux pattern as well as the mechanical properties, in particular, the electromechanical coupling factor, *k*. This factor is very important for sensing applications. Symmetry is also influential to properties through tensor coefficients. Final properties of the composite are evaluated using geometrical and arithmetical means of the properties of corresponding constituent phases. Newnham introduced the concept of connectivity in the piezocomposites in 1970s. Since then, it has grabbed much of the researchers’ attention. Out of the ten possible connectivity patterns (0-0, 0-1, 0-2, 0-3, 1-1, 1-2, 2-2, 1-3, 2-3 and 3-3), 0-3 and 1-3 are most widely used. Here, the first digit denotes the physical connectivity of the active phase of ceramic and the second digit refers to the passive phase of the polymer. For example, the (1-3) composite gives the one-dimensional connectivity of ceramic versus the three-dimensional connectivity of the polymer matrix. Employing of organic or polymeric polar or conductive components to the composite material results in improved electromechanical properties. A polymer may or may not exhibit piezoelectricity. Materials such as PDMS, SU8, and PVC which do not have any inherent piezoelectricity, but, because of their low stiffness coefficient, low acoustic impedance thermal and chemical stability, these materials can be combined with piezoelectric materials like BaTiO${}_{3}$ and PZT, which have high dielectric permittivity. Composites are excellent choices for having mechanical flexibility with a piezoelectric effect at low cost. Another type of composites can be made by mixing piezoelectric polymers such as PVDF and P(VDF-TrFE) with piezoelectric ceramics like BaTiO${}_{3}$, PZT, ZnO and SbSI. Here, the piezoelectric effect of polymer is further improved by incorporating piezoelectric ceramic particles. Such functionalized composites are ideal for nano power generation and bioimplantable applications. Piezocomposites, such as PZT/polyurethane have significant contribution in ultrasonic transducers, in terms of achieving complex shapes for focusing acoustic beams and low cross talk without cutting the transducer [15].

#### 3.2.3. Voided Charged Polymers (VCP)

VCPs, also referred to as cellular polymers or *piezoelectrets* which have the potential to exhibit higher piezoelectric constant than piezoelectric ceramics, are another interesting application of polymer thin films. Gas molecules in the thin films are ionized using electric field. This causes the opposite charges to accelerate and implant on either side of the voids, forming internal dipoles. Any deformation of the void can induce a piezoelectric effect in the material. The density, shape of the void, type and pressure of the gas are guiding factors for piezoelectric coefficient [16]. The early work by G. Dreyfus and J. Lewiner identified all the ingredients for piezo- and pyroelectricity in VCPs, such as nonuniform charge distribution, nonuniform strain, and suitable coupling between the two layers. These factors directly affect the net dipole moment created and the charge distribution on either side of the voids inside the polymer. Polymers used for VCPs must contain voids like foam, and should be electrically stable to trap and hold the electrical charges. Charge trapping can occur between electronegative groups on the chain or cages between adjacent molecules or at interfaces between crystallites and amorphous surroundings. In the VCPs, the piezoelectric coefficient can be varied with frequency of electrical field which gives us two different piezoelectric coefficients, namely *Quasistatic* and *Dynamic* [17]. The quasistatic piezoelectric coefficient is for zero or low frequencies, whereas the dynamic piezoelectric coefficient is for above 100 Hz. A thermodynamic layer model of a piezoelectret indicates that $d_{33}$ is directly proportional to the compressibility and inversely proportional to the foam’s Young’s modulus [18].

VCPs, owing to their low mass, high flexibility and almost arbitrary shape, are very much adoptable in measuring movements in biomedical applications, such as breathing movements of laboratory animals, respiration detector for humans and dynamic-force measurements on the limbs of running dogs [19].

### 3.3. Materials and Fabrication

#### 3.3.1. Bulk Polymers

##### Semicrystalline Polymers

Among other ferroelectric semicrystalline polymers, PVDF is the most prominent and widely explored piezoelectric polymer because of its fast-electro-mechanical response, high mechanical and chemical stability, flexibility, and low acoustic impedance. PVDF and its co-polymers like P(VDF-TrFE) are semicrystalline polymers which exhibit excellent piezo and ferroelectric properties. PVDF has five crystalline phases—α, β, γ, δ and ϵ of which α and β are the dominant ones [20,21]. The α phase being the most stable phase has trans-gauche-trans-gauche semi-helical conformation that develops upon cooling from melt. β-polymorph is polar and electroactive relevant in context to piezoelectric properties. β-phase exists in trans-conformation i.e., Hydrogen and Fluorine atoms are on the opposite side of the main backbone chain, resulting in non-zero dipole moment in PVDF. The P(VDF-TrFE) copolymer is formed by adding trifluoroethylene (TrFE) units to PVDF. P(VDF-TrFE) copolymer can directly crystallize into crystal structure of PVDF i.e., β-phase by stabilizing the all-trans chain conformation after introducing TrFE units to the PVDF which improves the piezoelectric effect. However, it is important to note that the dipole moment of the PVDF chain is reduced by the TrFE units because of the introduction of a third fluorine atom per repeat unit. Parylene-C is another piezoelectric material with single chlorinated benzene ring and has huge potential for bioMEMS application by virtue of its attractive mechanical properties and chemical inertness.

Unlike cumbersome and high temperature manufacturing processes involved in inorganic piezoelectric materials, piezoelectric polymers fabrication follows easy processes. Solvent casting is a simple process to make thin films by mixing and stirring under heat. For example, PVDF dissolved in solvents like, N-dimethylacetamide (DMA) and DMF, is poured on a glass mould to get the desired shape and size which is heated further in an oven. Spin coating is widely employed to get PVDF thin films.

Nano fibers produced using electrospinning of polymers is gaining popularity for its varied range of properties and applications. PVDF nanofibers have shown better performance than filled composites in terms of dielectric and piezoelectric constants [22]. Electrospinning also induces β-phase in PVDF and many groups are working on electro spun PVDF fibers. Vacuum vapor deposition is the common method used in fabrication of parylene thin films. The steps involved are achieving gas phase dimers of polymers like di-para-xylylene (DPX) using sublimation, creating monomer diradicals applying pyrolysis and finally polymerization on different substrate surfaces [23].

**Figure 3:**
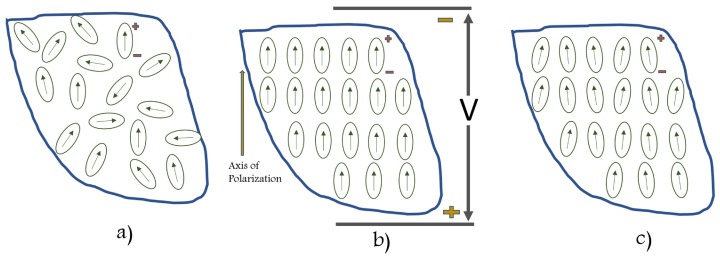
Poling process: (**a**) prior to polarization polar domains are oriented randomly; (**b**) a very large DC electric field is used for polarization; (**c**) after the DC field is removed, the remnant polarization remains.

Two important steps are involved for improving the piezoelectric effect in semicrystalline polymers: stretching and poling.They are explained in the following. 

(A) stretching:

Crystalline phase transformation is important for having piezoelectricity in semicrystalline material. Mechanical orientation, thermal annealing and high voltage treatment all can effectively induce the required crystalline phase transformations. Stretching the polymer essentially aligns the amorphous strands in the film in the planar direction and makes easy for the uniform rotation of crystallites by an electric field at later stages [24]. The electrical and mechanical properties (sensing direction) depend on whether stretching is uniaxial or biaxial. The orientation caused in the polymer chains in a specific direction by stretching results in orthotropic material in the piezoelectric sense. For small strains, however, the material is considered mechanically isotropic [25]. For example, a regular PVDF film will have high α- phase PVDF structure which has a zero-net dipole moment in the crystalline region. α-phase PVDF crystalline regions will align such that all dipole moments cancel each other out. On the other hand, the content of β-phase PVDF structure has fluorine on the one side and hydrogen on the other side with a net dipole moment in a stacked direction. β-phase can be increased using stretching. This means that stretching enhances piezoelectricity.

(B) Poling:

Above Curie temperature, $T_{c}$, perovskite crystal exhibits simple cubic symmetry with no dipole moment which means no piezoelectricity. However, the tetragonal symmetry associated with dipole moment returns below $T_{c}$. Alignment of the domains (regions of adjoining dipoles) provides with a net dipole moment thus a net polarization. As shown in Figure 3, direction of polarization among neighboring domains is random leading to zero overall polarization. A strong DC electric field at a temperature slightly below $T_{c}$ helps in aligning the domains in piezoelectric material. This is referred to as the “electrical poling”. Domains which are in close alignment with an electric field expand at the expense of domains that are not aligned with the field during poling in the direction of the applied electrical field. Most of the dipoles are locked into a configuration of near alignment, with an elongation, causing a permanent polarization called remnant polarization. The poling results in an increase in the length of the element which is very small, usually within the micrometer range. Typical values of applied electric fields are in the order of 50 MV/m which are sufficient to affect crystalline orientation.

**Figure 4:**
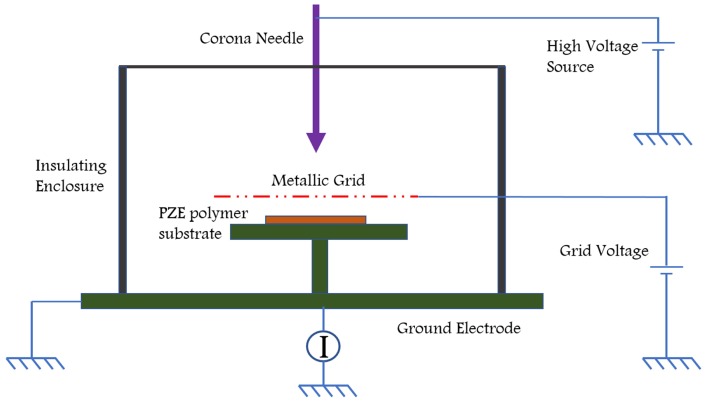
Schematic representation of a Corona poling station.

In the case of piezoelectric composites, ceramic particles need alignment using poling which can substantially increase the piezoelectric charge coefficient of the polymer matrix [14,26]. Poling is compulsory for these materials to exhibit piezoelectricity because of the low dielectric constant of the polymers. An applied electric field has a direct effect on the final piezoelectric constants obtained. The piezoelectric coefficient saturates after a specific value of electric field. Time of poling and temperature are crucial factors in modulating the dielectric constant of polymer. Two types of polymer poling methods can be adopted: (1) using a direct contact method or (2) corona discharge. For direct poling, electrodes need to be deposited on the sample and a high voltage is applied across the sample subsequently in a vacuum or insulated chamber. This is needed to avoid the dielectric breakdown of the sample. Typical corona poling set up is explained in Figure 4. The sample is placed on a ground plate and a high voltage (8–20 kV) needle is placed on the top at a distance (gap). A grid voltage may be employed for having uniform distribution of high voltage over large samples. Corona poling method is having an advantage as large area samples can be poled without the use of contact electrodes and can avoid dielectric breakdown of the sample. The medium in the chamber could be vacuum, air or insulated oil. Ionized gas particles around the needle are accelerated and bombard towards the sample surface, causing electric field across the substrate. Often conductive fillers like carbon nanotubes (CNT) are used in the samples to improve the dielectric constant. This gives a better poling effect [27]. Electrical poling is the common technique used to manufacture a commercially available PVDF film. Other interesting techniques such as self-poling have been adopted to align the dipoles in PVDF based polymers without any application of an electric field [28].

Amorphous Polymers: Polyimides are one of the most important amorphous polymers which can operate in harsh environments. Though they possess moderate piezoelectric voltage and strain coefficients, their ability to retain polarization at higher temperatures (up to 150 ${}^{\circ }$C) makes them interesting for high temperature applications. To improve the inherent piezoelectricity various methods like partial curing, corona poling are employed [27]. Aromatic polyimide like (β-CN) 1,3-bis-(3-aminophenoxy)benzene/1,3-bis-(3,4-dicarboxyphenoxy) benzene) (APB/ODPA) shows good performance in high temperature MEMS applications like pyroelectric sensors. High temperature tactile sensors are also developed using polyimides owing to their high glass transition temperatures [29].

Other amorphous polymers that find useful applications in piezoelectric sensors are polyacrylonitrile (PAN) [30], poly (vinylidene cyanide–vinyl acetate) (PVDCN/VAc) [31], poly(phenyl ether nitrile) (PPEN) and poly(1-icyclobutanecarbonitrile) [32].

#### 3.3.2. Polymer Composites

Low stiffness and flexibility of the polymers encourage researchers to use piezoelectric ceramics as filler material for tailor-made applications such as sensors and actuators. These filler materials are largely from piezoelectric ceramics such as BaTiO${}_{3}$, PZT, PMN-PT, ZnO, AlN. Owing to its high piezoelectric performance, PZT and their nano particles are widely experimented on combining with polymers like PDMS, PVDF, epoxies and resins. An important thing to observe is very high PZT content in the composite, sometimes, is not in favor of targeted mechanical properties. Additionally, lead based ceramics are not advised in bioimplantable applications and even has some serious environment concerns. Many new alternatives to lead are being developed such as LiNbO${}_{3}$ and KNaLiNbO${}_{3}$ [33], h-ZnO [34]. One of the widely used polymer matrices are polydimethylsiloxane (PDMS) and its copolymers which are soft materials with low stiffness coefficient. They must be chemically crosslinked to obtain free-standing films with very good mechanical properties for actuator, sensor and bio applications. However, since non-polar PDMS has a low dielectric permittivity, they are filled with ceramic particles with high ϵ. Polar silicones (chemically altered PDMS) are good candidates with high dielectric permittivity and inherent piezoelectricity as well. Carmen et al. made polar PDMS by attaching CN and Cl to PDMS [35].

The addition of fillers in the composites results in agglomeration of the fillers. Additionally, instability of the fillers in the polymer during processing can increase the dielectric losses. Aepuru et al. reported a user-friendly chemical process for the preparation of the filler that resulted in stable fillers in polymers and enhanced the dielectric properties of the composite at different frequencies and temperatures. They modified ZnO with polyaniline (PANI) as an interface in ZnO and used the PANI modified ZnO as fillers in PVDF [36].

**Figure 5:**
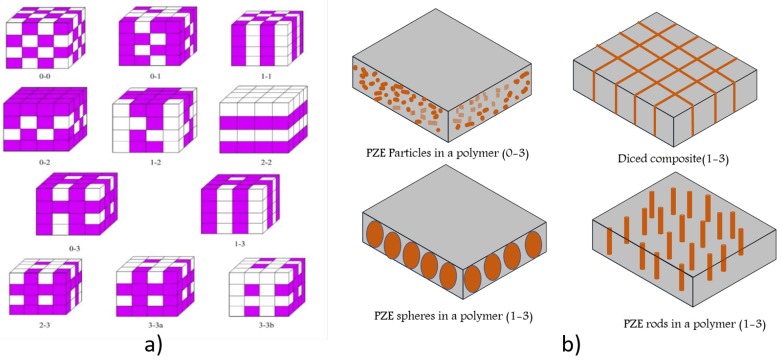
(**a**) Connectivity patterns for piezoelectric ceramic and polymer composites. Reprinted with permission from [40] © 2012 Elsevier. (**b**) Connectivity of piezo composites.

0–3 composites claim to be the simplest of manufacturing techniques among other connectivity patterns. Though they are simple, uniform dispersion of ceramic particles in the polymer and presence of voids are the important challenges faced. These voids may lead to low dielectric strength of the composite. The dice and fill method is used to achieve various connectivity patterns like 2-2 and 1-3, in piezoelectric composites. Low-cost nano structures can be developed using the lost mold method. Tape casting is extensively used for 2-2 connectivity with stacked tapes. The hot pressed method helps in well mixing of polymers and piezoelectric ceramics to produce shapes like discs and pellets by application pressure and temperature. Figure 5 describes the possible combination of connectivity patterns of piezo composites in practice [37]. A low cost molding process was used by Lee et al. to develop a micro structured PDMS which displayed high $d_{33}$ of 350 pC/N [38]. Woongchul et al. made thin films of BaTiO${}_{3}$ and PVDF by solution casting [39]. Herbal based ZnO mixed with PDMS was fabricated by Akakanksha et al. to demonstrate a piezoelectric material for soft touch applications [34]. Herbals are used as reducing agents for h-ZnO. Such applications are motivation for researchers to develop more eco-friendly piezoelectric materials.

#### 3.3.3. Voided Charged Polymers

Soft and high piezoelectric constants of piezoelectrets are important for water and air-coupled transducers with better acoustic impedance matching. Polypropylene is one of the pioneer materials used in the piezoelectrets. Piezoelectricity and pyroelectricity found in polypropylene space-charge electrets are explained by two near-surface charge layers of opposite polarity and by non-centrosymmetric trapping sites, respectively [41,42]. Good piezoelectric properties of porous polytetrafluoroethylene (PTFE) films and nonporous fluoroethylene propylene (FEP) have been already reported [43]. Recently, VCPs are reported using PEN (Poly ethylene napthalate) films with diffusion of CO${}_{2}$ gas to form voids [44]. Molded PDMS based VCPs are also reported [38].

In realization of VCPs, there are two major manufacturing steps involved. The first one is the formation of gas voids and the next one is the modification of gas voids for higher charge densities leading to superior piezoelectric response. By gas injection, spherical voids with nano or pico sized diameters are generated into the polymer melt. During the film blowing, the melt is extruded into a tube which is cooled down and reheated later. Degree of crystallinity highly depends on the draw ratio during extrusion. Typical gases used to create voids are air, N${}_{2}$ and CO${}_{2}$. Blow-extrusion process is majorly employed in producing cellular polypropylene VCPs [45]. In another method, high pressure gas, such as nitrogen, is used to uniformly saturate the polymer thus forming cellular structures. After this, by increasing the temperature and decreasing the pressure, permanent viscose changes in the polymer structure are achieved [46]. Controlling cavities inside these polymers while manufacturing is crucial to have uniform and better electrical charge distribution which influences resonant frequency. Thermo-forming methods are applied to achieve controlled geometrical cavities. Recently, 3D printing methods are employed to produce piezoelectrets with explicit structures and to study the piezoelectricity with different geometrical cavities with precise control [47].

Mohebbia et al. have synthesized a polymer electret using PP foam by saturating with inert gas in supercritical state and dropping the pressure. A high aspect ratio PP nano structures have been created by deploying co-extrusion with supercritical N${}_{2}$ using CaCO${}_{3}$ as a nucleating agent [48].

As discussed before, there is a large number of piezoelectric polymers. However, for real applications, there are some important factors and concerns that should be taken into account. Those factors are lifetime of the application, minimum response required for the application, tunability of mechanical properties and minimum level of required integration. The minimum response required is related to the coupling efficiency of the material. The higher the coupling coefficient, the higher the response is. Additionally, dielectric loss and mechanical loss should be taken into account for coupling efficiency as well. Some applications need a high temperature operation for a longer time. These applications need materials with high Curie temperature and less aging effect. The level of integration is related to the flexibility of fabrication processes of the materials. VCPs have highest piezoelectric coefficient compared to other piezoelectric polymers. However, due to low effective Young’s modulus, they have a low coupling coefficient. They also have uncertainty of lifetime of trapped charges and thermal stability. Among bulk piezopolymers PI (β-CN), APB/ODPA and PVDF are the best choices. PVDF and PI (β-CN) APB/ODPA have the same range of coupling efficiency. However, PI (β-CN) APB/ODPA has the highest operating temperature of all polymers. Bulk polymers such as PVDF and Paralyne-C are useful where impedance matching of acoustic sensors is critical for different media. Due to low Young’s modulus and moderate piezoelectric coupling coefficient, piezocomposites have benefits for sensing and energy harvesting applications. Their maximum operating temperature is determined by the glass transition temperature of the polymer used. Thus, using a polymer with high glass transition temperature is required to use them for high temperature operation. Electrodes are another important element, in the sensors, which carry the piezoelectric response to the measuring system. Copper tapes, conductive epoxies, physical deposition of metals such as silver, aluminum, and copper, printed conductive inks such as silver, graphene, CNT and poly(3,4-ethylenedioxythiophene) polystyrene sulfonate (PEDOT:PSS) have been tested as electrodes on piezoelectric polymers. An interesting thing to note is that Sampo et al. found a difference in piezoelectric response with the electrode material and CNT based ink providing the best sensitivity on PVDF substrates among other inks [49].

### 3.4. Recent Developments

Most recent developments of piezoelectric polymers and their applications are listed in Table 3. The intention of this section is to give the reader a overview of the performance of different piezoelectric polymers with more focus on application and manufacturing aspects.

## 4. Paper Based Substrates

### 4.1. Introduction

**Figure 6:**
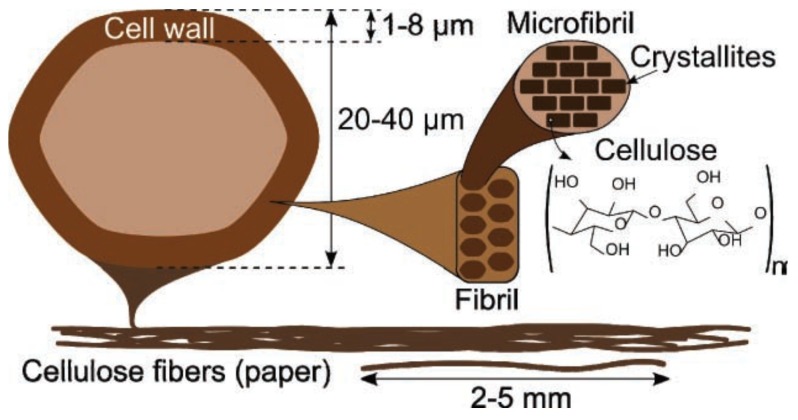
Cross section of cellulose fibers along with cell wall constructional details. Reprinted with permission from [65] © 2011 Wiley online library.

Paper is a ubiquitous material in the world directly associated with everyday use. It is one of the oldest and greatest invention by far to find many applications even today and most importantly it is environmentally friendly and biocompatible. Coming to electrical engineering, until recently, paper was regarded as an insulator for applications such as capacitor and high voltage insulation. After Bazhenov pioneered in piezoelectricity in wood fibers in 1950, a new paradigm was created for paper applications in the sensor industry. The converse piezoelectric effect in paper was discovered by E. Fakuda subsequently [58]. In 2000, Kim et al. demonstrated paper-based actuator and named it electro active paper (EAPap) [59]. A combination of ion migration and piezoelectric effect were employed to realize EAPap. The Whitesides group introduced microfluidic paper-based analytical devices (μpads) to build biosensors [60] in 2007. Inventions like these are propelling factors for the use of paper in sensor applications. Another significant development is blending materials like multi-walled carbon nanotubes (MWCNT), conducting polymers and ionic liquids into paper to realize hybrid paper actuators [61]. Some of the interesting applications developed in recent times using piezoelectric papers include rectenna [62], touch pads [63], and nano generators [64]. Paper is produced by hard pressing wood fibers called cellulose fibers which is an abundant material on earth. An important feature of paper is its tunable chemical properties, such as hydrophilicity, permeability and reactivity. It can be made from two types of pulps: chemical and mechanical pulps. Chemical pulp which is wood free yields a hygroscopic paper with long cellulose fiber and less lignin, while the mechanical pulp gives a paper with shortened cellulose fibers with amorphous lignin and hemicellulose. Structural details of paper based on wood cells (mechanical pulp) are furnished in Figure 6. Hemicellulose and lignin occupy the amorphous part of the fibrils (cell walls in the cellulose fibers are made up of fibrils) while the former is crucial for hydrophilicity and the latter accounts for hydrophobic nature of paper. Cellulose consists of glucose–glucose linkages arranged in linear chains. These fibrils are constructed by microfibrils in which crystalline regions are filled with cellulose chains held together by hydrogen bonding. Alignment of cellulose fibers highly influences the electrical properties of paper and can make it anisotropic or piezoelectric. Piezoelectricity in cellulose is due to dipolar orientation and monoclinic crystal structure of cellulose [61]. Additionally, electric and magnetic fields are employed for further enhancement in the piezoelectric effect.

### 4.2. Electroactive Paper (Eapap)/Smart Paper

Using paper as a smart material for sensor and actuator applications is known as electro-active paper (EAPap). EAPap [5] was discovered by Kim et al. in 2000. Actuators were fabricated with the cellulose paper which is coated with thin metal electrodes on both sides. EAPap mechanism is basically due to ion migration and piezoelectric effect associated with dipole orientation combined. Numerous advantages of EAPap are listed, such as being lightweight, dryness, low cost, biodegradability, large deformation, low actuation voltage and low power consumption [66]. Chemicals like poly(ethylene oxide)-poly(ethylene glycol) (PEO-PEG) improves the actuating mechanism of EAPap significantly. In the electrical perspective, the main advantages of EAPap over the piezoelectric polymers are that no electrolyte is needed for activation, it consumes very little power and has low activation voltage (0.25V/μm) [67]. The achieved piezoelectric coefficient is close to PVDF [59]. The ion migration effect in the cellulose can be improved by utilizing conducting polymers and ionic liquids as nano coats on the cellulose film, called *hybrid cellulose EAPap nanocomposite*. Polypyrole and Polyaniline (PANI) conductive polymer coatings are employed to achieve durable bending actuation in an ambient humidity and temperature condition [61].

A wide variety of applications are found using EAPap. A good review done by Asif et al. is found at [68]. Yuna et al. developed a EAPap actuator used for haptic sensing. They stretched the film at an orientation angle of 45${}^{\circ }$ to achieve a higher $d_{33}$ of 150 pm/V [69]. A cellulose paper-based audio speaker has been developed by Kim et al. by mixing cotton pulp with LiCl/N [70]. Ionic liquid and conductive polymer nanocoated cellulose EAPap have been developed to improve the performance of actuators in a humid atmosphere. Displacement results of nanocoated-actuators are far better compared to normal cellulose EAPap actuators under various RH conditions [71]. At low frequency strain measurements, EApap has good sensitivity in comparison with PVDF.

### 4.3. Hybrid Paper

Hybrid paper is a good alternative to piezoelectric polymer substrates, comparatively cheaper and environmentally friendly as well. This paper can be realized in multiple ways. One of the approaches is to incorporate the nanostructured piezoelectric material (ceramic) into wood cellulose fiber prior to the paper-making process—for example, by creating the opposite charges on functionalized wood fibers and BaTiO${}_{3}$ nano particles and attaching them at later stage [72]. This method claims to be simple, low cost and can be readily adapted by existing paper making processes. Here, piezoelectric properties of the paper are a direct function of BaTiO${}_{3}$ loading (ratio) and can be increased with loading. Piezoelectric paper made using this method has piezoelectric coefficient of 4.8 ± 0.4 pC/N) with 48 wt % of BaTiO${}_{3}$.

**Figure 7:**
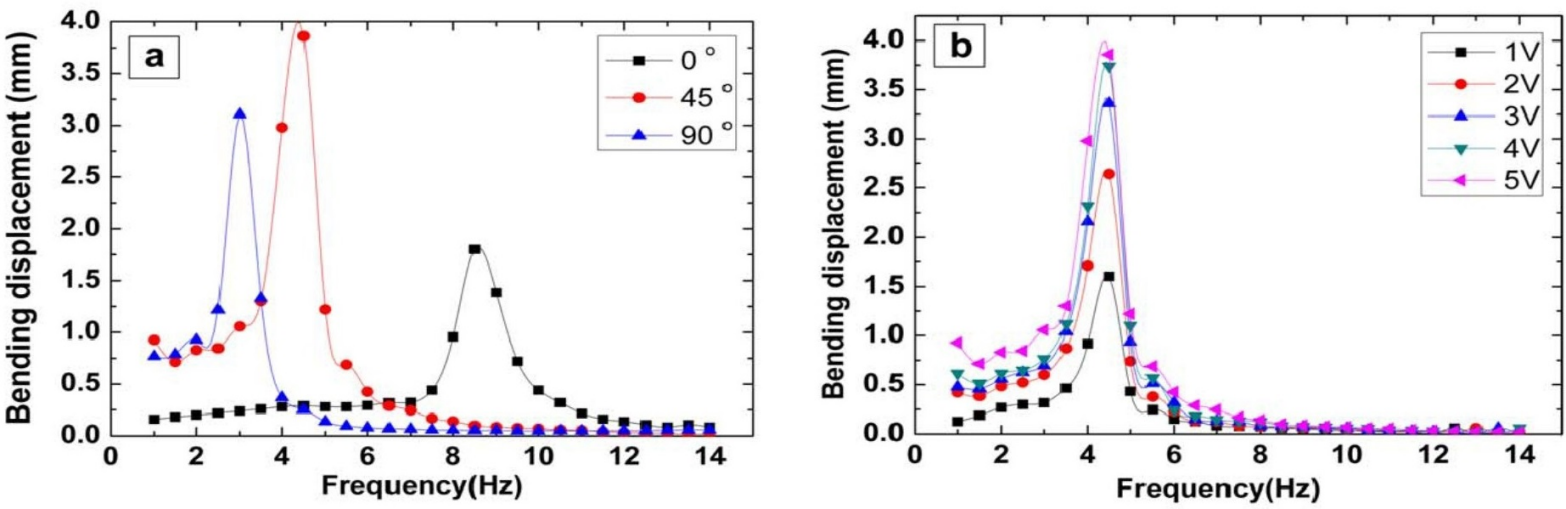
Bending displacement of piezo-paper actuator for different (**a**) orientation angles and (**b**) applied voltages. Young’s modulus varies with the orientation angle. Reprinted with permission from [61] © 2010, MDPI.

Another hybrid paper is produced by covalently grafting MWCNTs to cellulose (M/C) for the hybrid nanocomposite. A homogeneous distribution of MWCNTs in a cellulose matrix is achieved by a covalent grafting method [73]. The layered structure of cellulose matrix is distributed due to the effect of hydrogen bonds in cellulose improving mechanical strength of the composite fibers. Furthermore, the aligning of wood fibres is enhanced by mechanical stretching. This improves mechanical and electrical properties. The increase in Young’s modulus (23.4 GPa) and electrical resistance (107.7 kΩ) are indicative for this effect. The performance of a piezo-paper measured at different orientation angles (with respect to the stretching direction) is shown in Figure 7. The best performance is achieved with automated process and 45${}^{\circ }$ angle orientations.

### 4.4. ZnO NW-Papers

ZnO has a wurtzite structure with tetrahedral coordination of Zn${}_{2}$${}^{+}$ cations and O${}_{2}$${}^{-}$ anions [74]. The uniqueness of zinc oxide (ZnO) is that it exhibits multiple properties such as semiconducting, piezoelectric, and pyroelectric properties at the same time [75]. These features made them ideal for energy harvesting and sensor applications. ZnO nanostructures can be easily grown using a simple hydro thermal process at low temperatures, which is a huge advantage for researchers. By controlling the growth kinetics such as local growth temperature and the chemical composition, a wide variety of nanostructures can be produced. The growth can be restricted effectively to small areas, making it possible to realize functionalized sensors, such as touch pads, accelereometers, stress and strain gauges. In addition, by varying the aspect ratio, the piezoelectric coefficient can be tuned to specific needs [76]. While measuring the piezoelectric properties, it is important to eliminate the piezoresitive effect of ZnO nanowires which are bent during the contact force. In contrary to PZT and PVDF, ZnO has self poling ability by which electric poling can be avoided [28]. Using a solid-vapor phase thermal sublimation technique, a wide variety of modalities like nanocombs, nanorings, nanohelixes/nanosprings, nanobows, nanobelts, nanowires, and nanocages of ZnO are synthesized under specific growth conditions [74]. ZnO with multiple physical modalities are actively employed in paper-based substrates to develop some innovative applications. Conductivity and porosity of the paper are important to adhere to ZnO particles. High compatibility of ZnO with various paper substrates is encouraging the researchers in paper electronics.

A huge variety of piezoelectric applications are realized using ZnO nano structures on paper such as UV sensors, nano generators, accelerometers, strain sensors and so on. A touch pad based on ZnO NWs grown on paper has been demonstrated by Li et al. [63]. Gimenez et al. have developed a UV sensor on paper using ZnO band gap (3.3 eV) [39]. Wang et al. have used hydrothermally grown ZnO on cellulose chromatography paper to develop a one axis piezoelectric accelerometer [77].

### 4.5. Materials and Fabrication Techniques

Important factors to be considered for fabrication are simplicity, efficiency and low cost. Cellulose is the basic component and a wide variety of papers can be produced from it. Cellulose EAPap is made with a cellulose film called cellophane which is made with cellulose xanthate solution. This solution is made by saturating cotton pulps with sodium hydroxide. First, a solution is extruded through a nozzle into two sulfuric acid baths and then dried. When the solution coagulates in an acid bath, it converts back to cellulose. Incorporating some foreign particles such as BaTiO${}_{3}$, PZT into paper substrates to achieve piezolectricity has been explored. These are called “hybrid papers”, which have been discussed exclusively in the sections above. Preparation of hybrid papers such as cellulose fibers with piezoelectric ceramics involves activation of cellulose fibers using a positive electrolyte, followed by immersion of activated wood fibers in piezoelectric ceramic solutions (with negative electrolyte) to have a strong bond between the cellulose fibers and ceramic nano particles. An implantable power source in the human body is made by direct mixing of native cellulose with PDMS and MWCNT by a simple process [78].

ZnO and its hydro thermally grown nano particles are proven materials for piezoelectric papers used for touch and vibration sensing. Zno nano particle based paper fabrication involves hydrothermal growth of ZnO NWs. Performance of these devices hugely depends on different weight growth percentages of the ZnO NWs. The first step in preparing the ZnO based papers is to prepare the ZnO NPs solution followed by hydrothermal growth of ZnO NW’s on paper directly [63]. In another method, ZnO nano particles are mixed with custom-made inks and sketched on paper [79]. The reader can notice piezoelectric coefficients in some of the recently reported piezoelectric paper sensors in Table 4.

EAPaps have piezoelectric constants comparable to PVDF, low activation voltage and biodegradability. However, they do not have batch-to-batch consistency, have low carrier mobility and require toxic chemical processing. ZnO based papers employ simple manufacturing techniques, such as hydro thermal growth. However, in-homogeneous growth of nanowires and difficulty in selecting a suitable paper substrate with desired mechanical properties are the issues to use them on a large scale. Hybrid papers have shown good piezoelectric properties with simple processing and are suitable for energy harvesting applications. All of the piezo paper-based sensors developed so far does not match the performance of commercial devices and are not consistent with repeatability and scalability. Researchers are actively working on these issues. At the same time, the biodegradability and biocompatibility of paper is unquestionable, which is the motivation to use them for many applications.

## 5. Applications

**Figure 8:**
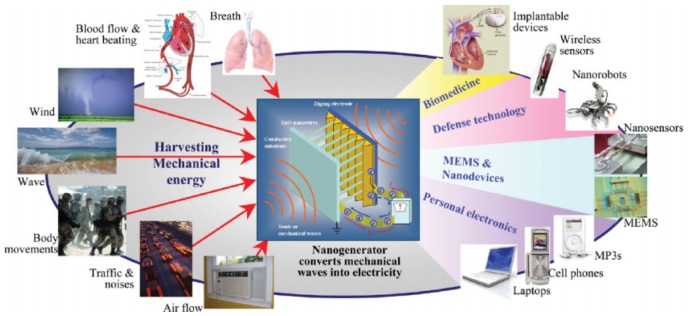
Possible applications of piezoelectric polymer/paper substrates; important areas are energy harvesting and sensing applications; Reprinted, with permission, from [84], © 2008 Wiley online library.

During the first world war, Langevin made the ultrasonic submarine detector which is the first application using piezo electric effect with the quartz crystal. More and more specialized devices have been made with the continued development of piezoelectric materials. These materials have set foot in many areas including sensors, actuators, resonators and energy harvesters and so on. Overall, these materials have endless opportunities. Advances in MEMS (Mico electromechanical systems) is the back end of many emerging piezoelectric applications like Lab-on-chip (LOC), Optical-MEMS (MOEMS), RF-MEMS, Power-MEMS and Bio-MEMS [83]. However, there are still some challenges such as fabricating electrodes for flexible substrates and thermal endurance of polymers and papers, which require researcher’s focus. In this section, we have tried to focus on recent interesting applications of piezoelectric polymers and paper in the sensor area. Possible areas of applications of piezoelectric paper and polymer are depicted in Figure 8.

Overall, we tried to focus on important areas such as tactile sensors, gas and VOC sensors, energy harvesting, bio and other sensors.

### 5.1. Tactile Sensors

A coordinated group of touch sensors gives rise to tactile sensing. Touch sensing essentially relates to the detection and/or measurement of a point contact force and location information. In everyday life, we encounter many such sensors such as elevator buttons and lamps that change their brightness after a touch. An important characteristic of these sensors is to measure very small changes in contact force with very high sensitivities. Tactile sensors are used in many applications like in the automobile industry, e.g., brakes, clutches, door seals, gasket, battery lamination, bolted joints, fuel cells and so on [85]. Piezoelectric polymer based tactile sensors can make a big difference in complex applications, such as robotic skin in terms of degree of system components, having a lightweight quality and flexibility. PVDF and its copolymer films have displayed a fast dynamic response in the 0–1 kHz frequency range which is necessary for robotic skin. Although tactile sensors can be based on different transduction techniques, such as resistive, mechanical and piezoelectric capacitive, the piezoelectric transduction technique has gained much of the researchers’ attention as they do not need any external power. Since most of these sensors require a soft piezoelectric material, the popular choices are PVDF, ZnO Nano wires and PZT in PDMS [86].

**Figure 9:**
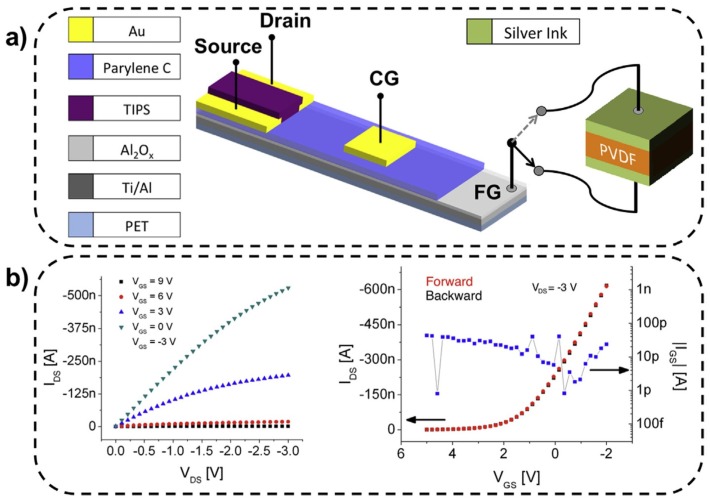
(**a**) Structural details of organic charge modulated FET (OCMFET) coupled to PVDF capacitor. (**b**) Output and input characteristics of OCMFET device for tactile sensing; Reprinted with permission from [87] © 2016 Elsevier.

**Figure 10:**
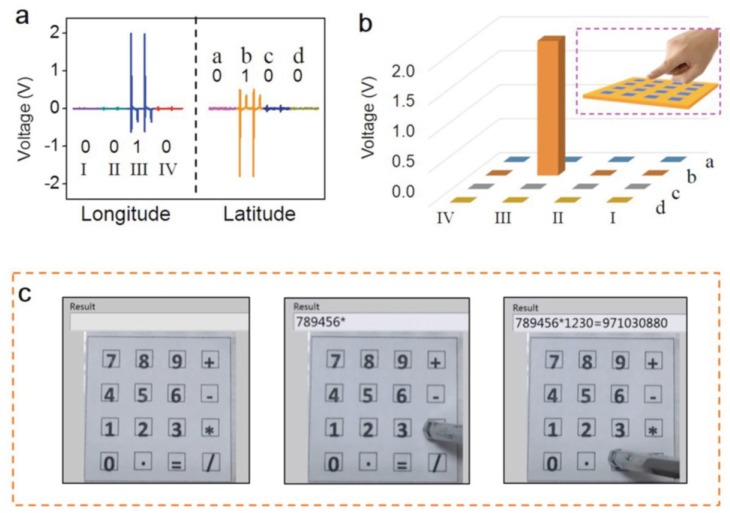
PATSA works as a calculator. (**a**) Longitude and latitude voltage data plots from the eight-channel electrodes of the PATSA when pixel (III-b) was subjected to a force.(**b**) Derived histogram sketch for this scenario. (**c**) Assembled flexible PATSA calculator. Reprinted with permission from [90] © 2015 Wiley online library.

An interesting application using an array of touch sensors has been developed for self sustainable e-skin or robotic skin [82]. Figure 9 shows a touch sensor developed by Spanu et al. using a PVDF capacitor with organic charge modulated FET (OCMFET), which can detect sensing pressures as low as 300 Pa in a wide frequency range (hundreds of Hz). No hysteresis and leakage current observed for the OCMFET [87]. A paper based tactile sensor (PATSA) developed by Zhong et al. for positions sensing is shown in Figure 10. Here, propylene piezoelectret is used as a sensing element, which is sandwiched between paper substrates. This sensor gives a sensitivity of 0.35 V/N.

In Table 5, the readers can find some recent developments in piezo polymer and paper based tactile sensors with their performance parameters. Electrode depositing and durability are challenges in manufacturing these type of sensors as they are subjected to intermittent mechanical stress.

Seminara et al. showed that ink-jet and screen printing methods employed to deposit electrodes provided better results [88]. A dome shaped PVDF touch sensor array was reported by Kim et al., which showed a better performance when compared to flat shape films. Patterning using SU8 polymer and air inflation techniques were used to have the dome shape [89]. Mahadeva et al. developed a tactile sensor with piezoelectric paper which was produced by blending cellulose with BaTiO${}_{3}$ particles [82].

### 5.2. Acoustic Gas and VOC Sensors

**Figure 11:**
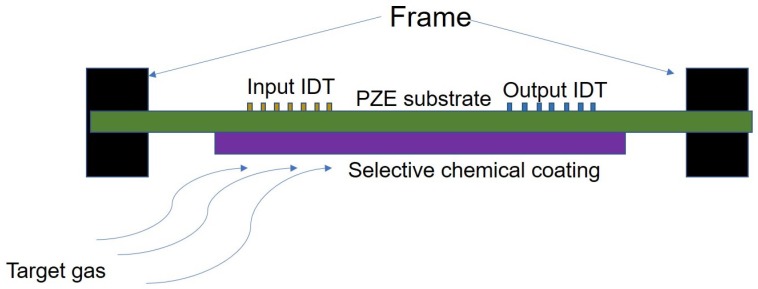
Cross-sectional view gas of sensor using a PVDF substrate [95].

In the beginning, acoustic wave devices were employed as bandpass filters in RF electronics. With the evolution of the technology, these devices found emerging applications in medical, automotive and industrial fields as well. Acoustic waves can change their resonanant frequency subject to the mass loading on the substrate [92]. The principle of an acoustic gas sensor lies in the interconversion of electrical and mechanical energies using piezoelectric substrate. This principle is extensively used for stress, strain, bio and gas sensing. For example, gas sensors utilize the mass sensitivity with the help of a selective chemical coating. This chemical coating absorbs the target gas and influences the mass loading of the sensor. Correspondingly, the mass of the target material deposited is predicted by measuring the change in resonant frequency of the device. A high electromechanical coefficient ($k^{2}$) and low temperature coefficient of frequency (TCF) are important parameters for acoustic gas sensors. Surface Acoustic wave (SAW), Flexural plate wave (FPW), and Lamb wave modes are being used to detect the various gas sensors [93]. SAW waves are widely applied to gas sensors, as most of the energy density is concentrated on the surface, which is important for detecting small amounts of absorbed gases. Influencing factors in acoustic sensing are piezoelectric constant, thickness of the substrate and effectiveness of sensing layer. Acoustic gas sensors are simple, low cost, compact, easy to deploy and ideal for sensing in remote areas. Additionally, they are low power devices and do not need a heating element like metal-oxide sensors to operate. Commercial gas analyzers with an array of SAW gas sensors are available in the market [92]. A good review of SAW based gas sensors has been done by Jagannath et al. [94]. Widely used substrate materials for these sensors are PVDF, cellulose paper. For electrodes PEDOT: PSS, copper, and aluminium are mostly used. Berring et al. reported a gas sensor in FPW mode to detect toluene vapor using a PVDF substrate, micro inkjet printed PEDOT: PSS polymer interdigitated transducers (IDT) appropriate and sensing layers [95]. Figure 11 explains the constructional details of toluene sensor by Berring et al. Polyvinyl acetate (PVA) and polystyrene (PS) as sensing layers to recognize varying concentrations of toluene vapor on a PVDF substrate.

### 5.3. Energy Harvesting

Wireless sensors are beginning to be deployed widely for many applications. A major issue with these sensors is powering them by batteries. Batteries have a limited life time and should be replaced regularly. Even rechargeable batteries have a limited duty cycle. Since the use of wireless sensors will grow in the future, researchers are considering energy harvesting by piezoelectric materials to be a sensible method for powering these sensors. One of the most abundant energies in the environment readily available for harvesting is mechanical energy, which ranges from mechanical vibration, wind energy to sonic waves, fluidics, bio motions, and more. Mechanical-to-electrical energy transduction mechanisms using a variety of piezoelectric materials are very popular and have been extensively investigated. In the last decade, there has been a surge in mechanical energy harvesting techniques using piezoelectric materials because of their ease of fabrication and wide-ranging applications. Many researchers succeeded in delivering interesting devices using them, such as self-powered wearable devices, nanogenerators and so on [84].

**Figure 12:**
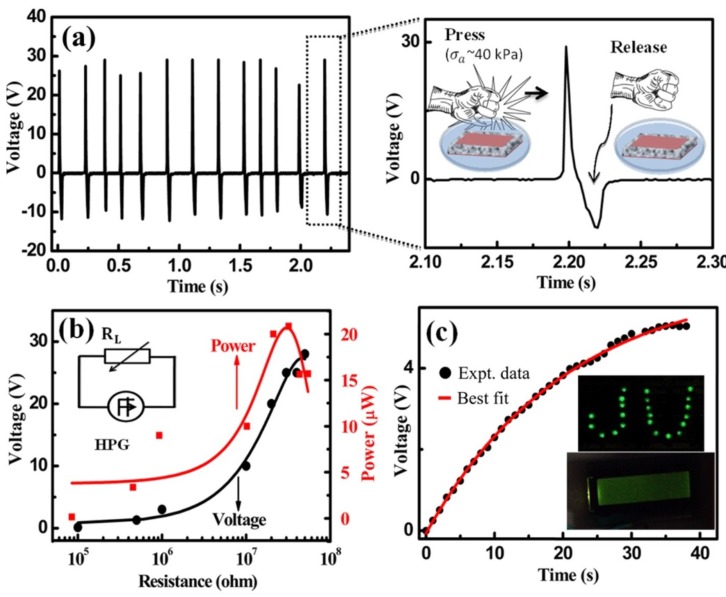
(**a**) Open-circuit output voltage of a PDMS/Cellulose/MWCNT based nanogenerator. (**b**) Voltage and instantaneous power change with respect to the load resistance (inset shows the corresponding circuit diagram). (**c**) Charging a capacitor from repeated human hand punching and releasing. LEDs and LCD screen (shown in inset of **c**) are lighted directly and from the charged capacitor, respectively. Reprinted with permission from [78] © 2016 ACS Publication.

Researchers tried blending polymers with many piezoelectric materials, such as ZnO, CdS, InN, ZnSn$O_{3}$, NaNb$O_{3}$, PZT, PMN-PT, BaTiO${}_{3}$, BiFeO${}_{3}$ and PVDF for fabrication of nanogenerators [55]. Owing to their high spontaneous polarization and low dielectric constants, BiFeO${}_{3}$ nanocomposites are suitable for vibrational energy harvesting. In addition, polymer nano composite (nPC) plays a predominant role due to their flexibility in operation. PZT, BaTiO${}_{3}$, P(VDF-TrFE) and cellulose paper are among the popular choices due to their encouraging results. PZT/polymer composites can realize energy harvesters with ultra low frequency and high power density that are crucial in self-powered MEMS devices. Recent developments in polymer and paper piezo nano generators (PNG) are reported in Table 6. Yanchao et al. reported a mesoporous PVDF thin film made with the help of ZnO nano structures. Kolev et al. have researched depositing the thin film of piezo materials using different methods such as spray coating and RF sputtering [96,97]. They prepared a flexible piezoelectric device for portable energy harvesting applications based on a nanobranched ZnO film deposited by RF sputtering on polymer/metal/polymer substrate [97]. This porous PVDF film has showed a very good open circuit voltage due to high β-phase content of porous structure [98]. Zhi et al. mixed PZT and PDMS and tape casted to develop a PNG that showed a maximum open circuit voltage of 8 V [99]. Bartlomiej et al. developed an antimony sulfoiodide (SbSI) based blotting paper PNG and showed a power density of 41.5 nW/cm${}^{3}$ [80]. The nano generator showed in Figure 12 was developed by Mehebub et al. using native cellulose micro fiber, PDMS and MWCNT with direct mixing. This has the potential to be a bio implantable power generator [78].

Electro mechanical coupling coefficient (k) and Electro mechanical transmission factor ($\lambda_{max}$) are important factors for piezoelectric energy harvesting appliactions [100] as they describe the stored and utilized energies.

### 5.4. Bio and Other Sensing

The bio sensing field is always in need of non-toxic, self powered, high resolution, light weight and reliable materials for sensing parameters of human body. A huge cross section of natural and synthetic materials with different molecular designs are studied for biomaterials for tissue and biomedical engineering in the recent past. Polymers from natural origin are often difficult to process and show poor desired mechanical and electrical properties. The use of inorganic-based flexible piezoelectric polymers and papers for biomedical applications have been actively reported due to their advantages of highly piezoelectric, pliable, slim, lightweight, and biocompatible properties [104]. They can convert tiny mechanical responses in the body into readable signals by electronics, and can even respond to small movements on corrugated surfaces of internal organs [105].

**Figure 13:**
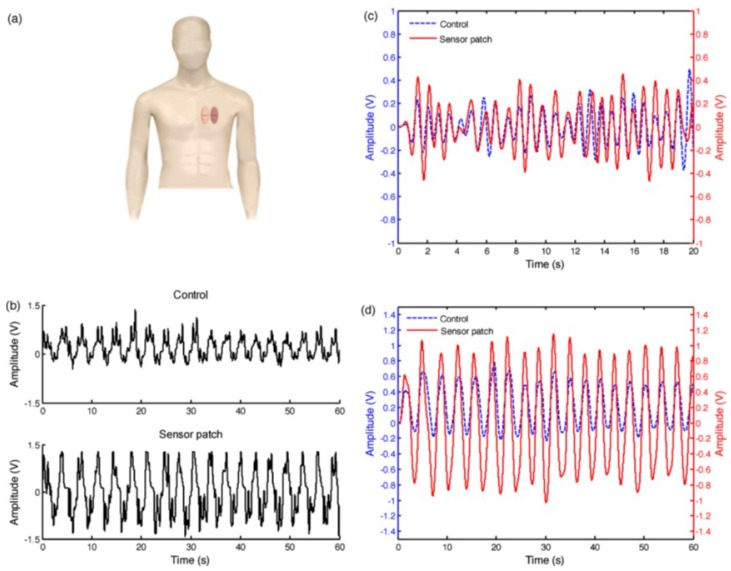
PVDF sensor for heartbeat and respiration detection. (**a**) Schematic representation of the adhesion of the PVDF sensor and control sensor patch on the chest wall of a human body. Comparison of the electrical signals obtained from the control sensor, and the proposed PVDF sensor: (**b**) raw electrical signals, (**c**) filtered signals for heartbeat detection, and (**d**) filtered signals for respiration detection. Reprinted with permission from [107] © 2013 Elsevier.

Piezoelectric micro-electromechanical systems (MEMS) resonant sensors are already applied in different sensing areas including tissue engineering, DNA hybridization, protein–ligand interactions, and immunosensors. The changes in resonant frequencies of piezoelectric polymers, such as PVDF are utilized in structural health monitoring applications. Tushar et al. reported a PVDF coated on silicon sensor for catheter application which can detect small pressure changes [106]. This application can help patients for effective blood transfusion. A sensor built using PVDF substrate by Chiua et al. to measure the pulsatile vibrations and periodic deformations on the chest [107] is shown in Figure 13.

**Figure 14:**
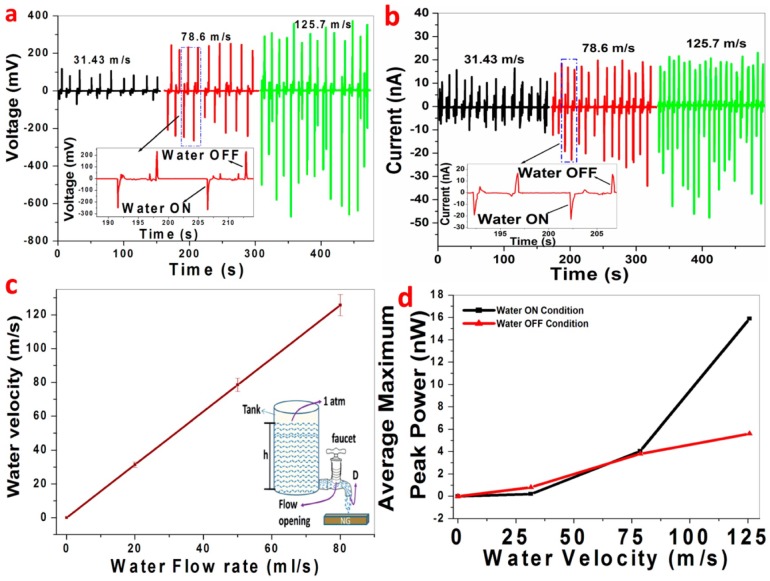
(**a**) Open circuit voltage and (**b**) short circuit current wave forms of the PVDF/BaTi${}_{1-x}$Zr${}_{x}$O${}_{3}$ based self-powered fluid velocity sensor for different water flow velocities with periodic ON/OFF conditions. (**c**) Linear relationship between water velocities and flow rate was obtained. The schematic diagram of the control experiment is shown in the inset. (**d**) The average maximum output peak power achieved for different velocities during ON/OFF conditions. Reprinted with permission from [109] © 2015 ACS publications.

Piezoelectric polymers and paper have made their presence in numerous other applications starting from strain, force and UV light sensors, accelerometer and so on. A strain sensor used in structural strain monitoring is devised by Hemtej et al. with plain printing paper and ZnO NWs [108]. A fluid velocity sensor reported by Naga malleswara rao et al. [109] is made of hybrid film with PVDF and BaTi${}_{1-x}$Zr${}_{x}$O${}_{3}$ films and has shown a linear response to water flow. The schematic is shown in Figure 14. Another important application of piezoelectric paper is for radio-frequency identification (RFID) tags developed in recent years.

**Figure 15:**
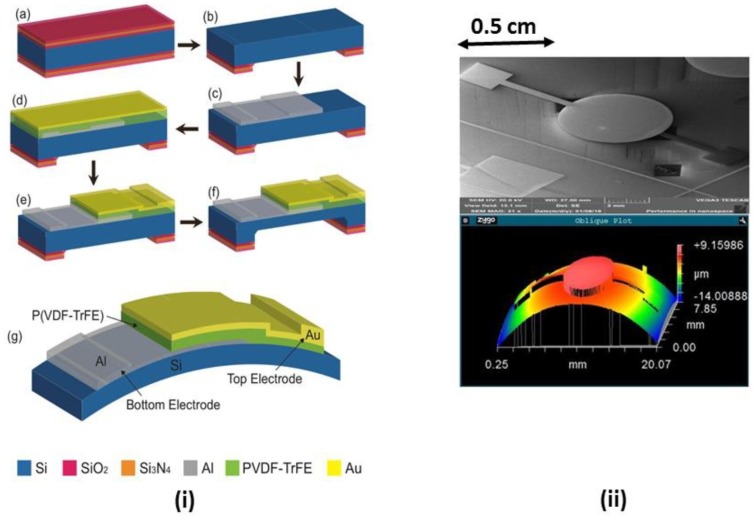
(**i**) Steps to fabricate ultra-thin silicon based PVDF-TrFE capacitors: (**a**) Hard mask growth (**b**)Backside patterning to open etching window (**c**) Bottom metal deposition and patterning (**d**) PVDF-TrFE spin coating, annealing and top metal deposition (**e**) Patterning top metal and dry etching of polymer (**f**) Wet etching of bulk silicon (**g**) Final device on thin silicon (**ii**) (**top**) Scanning Electron Microscopy image of the piezo-capacitor sensor (**bottom**) optical profilometer image showing warp image of thin chip with the piezo-capacitor. Reprinted with permission from [110] © 2016 Elsevier.

Gupta et al. developed a P(VDF-TrFE) capacitor based sensor on an ultra-thin silicon substrate that is shown in Figure 15 [110]. Wang et al. reported an accelerometer developed using ZnO nano wires thermally grown on U-shaped paper with a cantilever arrangement [77].

## 6. Conclusions and Future Scope

This review summarizes recent progress in piezoelectric polymers and papers. We have focused on numerous choices of piezoelectric polymers and papers with various materials and fabrication techniques available to prepare them. A wide range of possible applications specially for sensors has been discussed. Though these polymer and paper materials offer cost effective, simple solutions for many applications, they still suffer from few disadvantages, such as poor thermal stability, difficulty to fabricate electrodes, short lifetime and integration problems. Only a few of the piezo polymer and paper mentioned in the paper can withstand more than 100 ${}^{\circ }$C. Designing compatible and flexible electrodes for polymer and paper piezo substrates is another challenge. Some stretchable electronic applications need electrodes that are flexible to support up to two times the length of the film. Lead toxicity in piezo polymer and paper using PZT, PMN-PT, PbTiO${}_{3}$ is a limiting factor to use them in biomedical applications. Poor current supply capability is another impeding factor for these materials to be used as substrates while integrating on chip electronics. Researchers and engineers are actively working on these issues and some alternatives such as PVDF-BaTiO${}_{3}$, PZT-cellulose paper, cellulose paper-PZT are emerging. These materials have an edge over existing piezoelectric polymers and papers in terms of piezoelectric constants, thermal stability and lifetime. These substrates allowed researchers to think in new directions and allowed for the realization of innovative products like harvesting energy from movements of human body, pressure, flow, stress, strain, and force sensors. Other opportunities from these materials are in smart robotics and metrology tools. Finally, inspiration behind the advancement in piezoelectric polymers and paper should be using materials, fabrication techniques and device design mechanics that will make a cleaner, greener and better world.

## Figures and Tables

**Table 1:** Important material parameters for piezoelectric ceramic, polymer, and composites. Positive and negative signs next to the properties of ceramic and polymer show the advantages and limitation, respectively of that particular parameter.

Parameter	Ceramic	Polymer	Composite
Acoustic impedance	30 (−)	4.3 (+)	10 (can be tailored)
Coupling factor	0.5–0.7 (+)	0.1–0.3 (−)	0.5–0.7
Spurious modes	many (−)	weak (+)	weak
Dielectric constant	200–5000 (+)	8–10 (−)	proportional to vol% of PZT
tan $\delta_{m}$	0.0125 (−)	0.1 (+)	0.05
tan $\delta_{e}$	0.02 (+)	0.25 (−)	0.02
Cost and Ease of fabrication	cheap (+)	expensive (−)	medium

Reprinted, with permission, from [10] © 1992 IEEE.

**Table 2:** Physical properties of PZT (ceramic), PVDF (polymer), and Piezoflex and Piezel (composite materials).

Property	PZT (PC5)	PVDF	Piezoflex1 #	Piezel *
Density (kg/m${}^{3}$)	7750	1800	4500	5600
Sonic velocity (m/s)	2830	1400	2100	1687
Acoustic impedance (Mralys)	22	3	10	9.8
Compliance ($\times 10^{-9}$ m${}^{2}$/N)	0.02	0.1	13	0.25
Relative permittivity	1800	10	32	70
Tanδ (at 1 kHz)	0.02	0.05	0.08	0.047
$d_{33}$ (pC/N)	410	30	25	40
$d_{31}$ (pC/N)	−175	−18	−4.6	−24
$g_{33}$ (mV-m/N)	26	340	88	–

Reprinted, with permission, from [11], © 1994 Elsevier. # Piezoflexl PZT embedded in an inactive epoxy resin matrix with a volume distribution of 50–50%. * Piezel is PZT core embedded in a matrix of the PVDF with a volume distribution of 50–50%.

**Table 3:** Recent reportings on piezoelectric polymers.

Material	Reported ‘d’	Manufacturing Techniques	Applications
PVDF with Facile Phase Inversion Technique [13]	≈49.6 pm/V	(1) PVDF solution spin coated on glass substrate. (2) Self polarized films using phase inversion technique by varying quenching temperature.	Possible applications: Actuators and Energy Harvesting
AlN thin film sputtered on PET [50]	0.7 pC/N.	(1) Aluminum Nitride films on PET substrates using RF magnetron sputtering. (2) Platinum electrodes are deposited with same sputtering.	Measuring human pulse
PZT + PDMS Composite solution casted [51]	25 C/N	(1) Thermally treated PZT and PDMS mixed and solution casted on a polycarbonate substrate. (2) Poling at 12 kV/mm at 120 ${}^{\circ }$C	Possible applications: Tactile sensors
BaTiO${}_{3}$ NPs in PVDF [39]	61 pC/N	(1) Synthesized BaTiO${}_{3}$ nanowires mixed with PVDF solution and dispersed on glass substrates. (2) Poling at 30 MV/m at room temperature.	Possible applications: Actuators and Energy Harvesting
Micro patterned PDMS [38]	350 pC/N	(1) PDMS is spin coated on patterned molds. (2) Micro-structured PDMS were sandwiched between gold electrodes and charged by direct contact method.	Not reported yet
Poly Propylene extrusion with N${}_{2}$ [48]	250 pC/N	(1) Polypropylene foam films extruded with CaCO${}_{3}$ as nucleating agent (2) Stretched to form eye like structures and poling at 21 kV for 60 s.	Not reported yet
P(VDF-TrFE) Nano tube array with Al matrix [52]	−35 pm/V	(1) Vertically aligned P(VDF-TrFE) nanotube arrays embedded in Aluminum membrane matrix. (2) Piezoelectric voltage constant more than monolithic film.	Possible applications: Actuators and Energy Harvesting
PZT + Liquid Crystalline resin (thermosetting) [53]	$g_{33}$ = 48 mV-m/N	Liquid Crystalline resin (HBA-HBNA) and PZT powders are mixed above melting point casted films using two methods: cross linking and high temperature processes.	Possible applications: Pressure Sensors
P(VDF-HFP) porus thin films using ZnO-Nano particles [54]	−15.2 pC/N	(1) Self poled, ZnO nano particles etched, porus P(VDF-HFP) films made using casting. (2) ZnO NP’s are removed using HCl solution.	Tiny human activity sensor
BaTiO${}_{3}$ nanowires and PVC composite fibers [55]	13.7 pC/N	(1) BaTiO${}_{3}$ nanwires and PVC powder mixed and composite fibers are created by spinning. (2) Fibers placed on PET substrtate, coated with PDMS, cured and poled at 3 kV/mm at 100 ${}^{\circ }$C	Finger motion sensor
Herbal-ZnO + PDMS [34]	29.76 pm/V	(1) Herbals are used as reducing agents to synthesise h-ZnO. (2) h-ZnO and PDMS are mixed to prepare different ratio composites.	Soft touch applications
Electro spun PVDF—Ag functionalised CNTs composite fibers [22]	54 pm/V	(1) PVDF fibers with enhanced β-phase content by electrospinning. (2) Silver coated MWCNT functionalization to enhance the piezoelectric coefficient of fibers	Possible applications: Actuators and Energy Harvesting
Cyano attached PDMS [35]	47.6 pm/V	Cyanopropyl-modified polysiloxanes and Chloro-modified polysiloxane are crosslinked with non polar PDMS for producing all polymer polar silicones.	Tactile, pressure and acoustic sensors
KNLiNbO${}_{3}$ micro cubes + PDMS composite [33]	$d_{33}$ = 17 pC/N and $g_{33}$ = 220 mV-m/N	(1) Alkaline niobate and PDMS composites are fabricated by solid state sintering (2) Microcubes are aligned using dielectrophoresis by varying frequency of AC electric field.	Wireless sensors and biodiagnostics.
Polyetherimide + lead titanate (PT) composites [56]	$d_{33}$ = 7.2 pC/N and $g_{33}$ = 102 mV-m/N	Solution casting and Dielectrophoresis processing later results in construction of chain like structures of PT particles in the polyamic-acid matrix; Traditional poling below $T_{g}$.	Possible applications: Structural health monitoring, space applications, harsh environments
Acrylobutylnitrie piezoelectrets [47]	$d_{33}$ = 70 pC/N	3D Printing process performed by melting the polymer filament into layers for creating the void cells.	Possible applications: Water and air coupled transducers
Paylene-C films [23]	$d_{33}$ = −2.0 pC/N	(1) Deposition of thin film Parylene using vaporizing, pyrolysis and polymerization steps. (2) Poling conditions changed from 5 MV/m to 40 MV/m with temperatures 100 to 250 ${}^{\circ }$C	Possible applications:BioMEMS
Parylene (poly-p-xylylene, PPX) thin films [57]	$d_{33}$ = 200 pC/N	(1) Thin PPX films created by replacing conventional pyrolysis plasma decomposition using DPX precursor. (2) Achieved higher growth rates compared to conventional CVD.	Possible applications:BioMEMS

**Table 4:** Piezoelectric coefficients were reported in a recently developed piezo paper.

Material	Piezoelectric Coefficient	Applications
Stacked thin Cellulose film [69]	150 pm/V	Actuator/Resonator
BaTiO${}_{3}$ NPs in Wood Cellulose [72]	4.8 ± 0.4 pC/N	Potential Applications: Sensors and Actuators
SbSI NWs in blotting paper [80]	$1\times 10^{-9}$ C/N	Energy Harvesting
Rochelle salt impregnated paper [81]	3–25 pC/N	Potential Applications: Sensors and Actuators
BaTiO${}_{3}$ Nano structures in Wood Cellulose [82]	37–45.7 pC/N	Potential Applications: Energy Harvesting

**Table 5:** Observations of recent developments in Tactile Sensors

Materials	Sensitivity	Applications
ZnO NW with Cellulose paper [77]	16.1 mV/g	Vibration sensing in Smart packaging
Poly propylene-based Sensor array (PASTA) [90]	0.3 V/N	Touch sensors
P(VDF-TrFE) and Silver electrodes screen printed [91]	≈0.05 V/N	Touch sensor array
PVDF film with Dome shaped structure [89]	8.83 × 10${}^{-3}$ V/mN	Touch sensor array
PVDF film + OCMFET [87]	Can detect Pressure as low as 300 Pa	Touch sensor for e-skin

**Table 6:** Recent developments in energy harvesting.

Materials	Output Power Density/Short Circuit Current	Open Circuit Voltage	Thickness	Remarks
Native Cellulose Microfiber + PDMS + MWCNT [78]	90 μW/cm${}^{3}$	≈30 V for shock pressure 40 kPa	Not mentioned	
Paper + SbSI NW’s [80]	41.5 nW/cm${}^{3}$	2.4 V for shock pressure 3 Mpa, 24 mV for sound excitation of 90 dB	0.05 mm	
PMN-PT + MWCNTS in Silicone matrix [101]	500 nA	4 V	Not mentioned	Stretchable upto 200%
ZnO NWs + Paper [102]	51 μW/cm${}^{2}$, 35 nA	18 mV	Not mentioned	Suitable for Stretchable and Wearable electronics
P(VDF + TrFE) + Cellulose paper with Pt electrode [103]	2.85 mW/cm${}^{3}$, 0.38 μA	1.5V	1 μ	Wearable electronics
PZT NWs + PDMS [99]	2.4 μW/cm${}^{3}$, 175 nA	8 V	0.018 cm	Energy Harvesting
Mesoporous PVDF made with ZnO NPs [98]	0.16 mW/cm${}^{3}$, 9.8 μA	11 V	28 μ	Energy Harvesting
